# HIV incidence and prevalence among adults in Mozambique: estimates from the Population‐based HIV Impact Assessment Survey (INSIDA 2021) and district‐level modelling

**DOI:** 10.1002/jia2.70008

**Published:** 2025-10-28

**Authors:** Eduardo Samo Gudo, K. Carter McCabe, Erika Fazito, Daniel Catano, Orrin Tiberi, Makini Boothe, Jordan McOwen, Jeffrey W. Imai‐Eaton, Oliver Stevens, Lourena Manembe, Wafaa M. El‐Sadr

**Affiliations:** ^1^ Instituto Nacional de Saúde (INS) Maputo Mozambique; ^2^ Division of Global HIV & TB, US Centers for Disease Control and Prevention (CDC) Maputo Mozambique; ^3^ ICAP at Columbia University New York New York USA; ^4^ Programa Nacional de Controle de ITS‐HIV/SIDA, Direção Nacional da Saúde Publica, Ministério de Saúde Maputo Mozambique; ^5^ UNAIDS Maputo Mozambique; ^6^ Center for Communicable Disease Dynamics, Department of Epidemiology, Harvard T.H. Chan School of Public Health Boston Massachusetts USA; ^7^ MRC Centre for Global Infectious Disease Analysis, School of Public Health, Imperial College London London UK; ^8^ Conselho Nacional de Combate ao HIV/SIDA Maputo Moçambique

**Keywords:** HIV, incidence, Mozambique, Naomi model, prevalence, Population‐based surveys

## Abstract

**Introduction:**

Accurate information is needed to prioritize programmes and resources that address gaps in the HIV response. We examined findings from the 2021 Mozambique Population‐based HIV Impact Assessment (INSIDA) survey, complemented with subnational model‐based estimates of the number of new infections and district‐level incidence to gauge progress in the HIV response and guide future priorities.

**Methods:**

INSIDA 2021, a nationally representative cross‐sectional household survey, measured national HIV incidence, national and provincial HIV prevalence, and factors associated with HIV. Consenting adults aged 15 years and older were interviewed and tested for HIV using the national diagnostic algorithm, followed by laboratory‐based confirmation of HIV status. Testing for viral load, limiting antigen avidity and the presence of antiretrovirals were used to estimate HIV incidence. The Naomi model, a Bayesian small‐area estimation model combining the INSIDA 2021 survey and routine HIV service delivery data, estimated provincial and district‐level HIV incidence and district‐level prevalence. Weighted HIV prevalence estimates, stratified by sex, are reported and factors associated with HIV infection modelled via multivariate logistic regression.

**Results:**

National HIV prevalence was 12.5% (95% CI: 11.5−13.4) among adults 15 years and older, and national HIV incidence was 4.3 (95% CI: 2.3−6.3) per 1000 HIV‐negative adults in 2021. Per model estimates, there were 84,000 (95% CI: 80,000−89,000) new infections per year, 55,000 among women (95% CI: 52,000−58,000) and 30,000 (95% CI: 28,000−31,000) among men. In 2023, an estimated 2.2 million (95% CI: 2,200,000−2,300,000) adults (15+ years) with HIV were living in Mozambique. District‐level estimates highlighted areas of higher adult HIV prevalence and incidence in urban areas of key cities and ports, in the south, and along coastal districts in central Mozambique. Compared to men the same age, the distribution of HIV infections remains concentrated among women, particularly young women.

**Conclusions:**

Mozambique continues to face a high burden HIV epidemic, with high HIV incidence associated with spatial heterogeneity. Prevention of new infections through women and young women‐centred prevention programmes, treatment for men, and focusing interventions in urban areas, port cities, and coastal areas in central and southern Mozambique could contribute to reducing the HIV burden in Mozambique.

## 1 INTRODUCTION

Since the last national HIV survey in 2015, Mozambique has rapidly scaled‐up provision of HIV services, with the number of people on antiretroviral therapy (ART) increasing from 802,660 in 2015 to 1,402,900 in 2020 and expansion of combination HIV prevention—with 1.8 million voluntary male medical circumcisions (VMMCs) performed and 373 million condoms distributed between 2015 and 2019, increased HIV awareness among youth, and focused interventions for key populations [1, 2, 3, 4]. Thus, new national and subnational HIV estimates were needed to optimize programmatic focus and resource allocation.

The Mozambique Population‐based HIV Impact Assessment (INSIDA 2021) survey was conducted to evaluate progress in Mozambique's HIV response; however, it was not powered to provide district‐level HIV prevalence or subnational HIV incidence estimates. Therefore, survey findings were complemented with subnational HIV prevalence and incidence estimates obtained through the Naomi model, which derived estimates from INSIDA 2021 and routine programmatic data, to provide localized estimates critical for HIV programmatic planning. Using INSIDA 2021 data and Naomi estimates, authors present sex‐specific provincial and district‐level HIV incidence and district‐level prevalence estimates, and examine socio‐demographic and behavioural factors associated with HIV prevalence, among adults aged 15 years and older in Mozambique.

## 2 METHODS

INSIDA 2021 was a nationally representative, household‐based survey, conducted between April 2021 and February 2022 to assess Mozambique's national and provincial HIV response. Using a four‐stage probability sample design, 311 enumeration areas (EAs) were sampled across Mozambique's 11 provinces. Eligible individuals, aged 15 years and older who slept in the household the night prior, who provided written, informed consent, responded to a standardized demographic and HIV care and risk behaviours questionnaire. Trained phlebotomists collected venous blood samples from consenting participants and conducted home‐based HIV testing and counselling following national guidelines, which included a Determine™ HIV‐1/2 (Abbott Molecular Inc., Des Plaines, Illinois, USA) screening test followed by a Uni‐Gold™ (Trinity Biotech, plc., Wicklow, Ireland) confirmatory test. HIV‐test results were provided to participants the same day at the household, with appropriate counselling and referral.

Specimens testing HIV positive or indeterminate during household‐based testing were confirmed in the laboratory via Geenius™ HIV 1/2 Supplemental Assay (Bio‐Rad, Hercules, California, USA). A reactive Geenius result defined HIV‐positive status for survey results. Plasma or dried blood spots (DBS) from confirmed HIV‐positive participants were tested for viral load (VL) (HIV RNA copies/ml) via COBAS® AmpliPrep/COBAS TaqMan®. Qualitative screening for detectable concentrations of the antiretrovirals (ARVs) most commonly prescribed in Mozambique (atazanavir, lopinavir, efavirenz and dolutegravir) was performed at the University of Cape Town on DBS specimens from confirmed HIV‐positive participants using high–resolution liquid chromatography coupled with tandem mass spectrometry [5]. A laboratory‐based incidence testing algorithm (HIV‐1 limiting antigen [LAg]‐avidity enzyme immunoassay assay [EIA] with correction for VL and detectable ARVs) was used to distinguish recent from long‐term HIV infection among HIV‐positive samples. The Sedia HIV‐1 LAg‐Avidity EIA (Sedia Biosciences Corporation, Portland, Oregon, USA) was used on plasma specimens, while the Maxim HIV‐1 LAg‐Avidity DBS EIA (Maxim Biomedical, Bethesda, Maryland, USA) was used on DBS specimens when plasma was insufficient. Plasma specimens with median normalized optical density (ODn) ≤ 1.5 and DBS specimens with median ODn ≤ 1.0 were classified as potential recent infections, and their VL results assessed [6]. Specimens with VL < 1000 copies/ml were classified as long‐term infections. Specimens with VL ≥ 1000 copies/ml were assessed for the presence of ARVs: those with detectable ARVs were classified as long‐term infections, and those without were classified as recent infections [7]. Incidence was calculated using the World Health Organization recommended formula, with a mean recent infection duration of 130 days (95% CI: 118–142), a 1.0 year time cutoff and a percentage false recent of 0.00 [8].

The Naomi model was used to estimate provincial and district‐level HIV incidence, district‐level HIV prevalence, and numbers of people living with HIV (PLHIV) and new infections. Naomi is a Bayesian small‐area estimation model that estimates HIV indicators stratified by age group, sex, and district through statistical modelling of HIV prevalence and ART coverage data from nationally representative household surveys and routine district‐level HIV programme data—the number receiving ART, and HIV prevalence and ART coverage among pregnant woman attending first antenatal care visit [9]. The model incorporates programme data for the most recent survey year (2021) and the most recently available programme data (2023) [10]. HIV estimates presented here are from the 2023 Estimates using Spectrum version 5.29 and Naomi version 2.9.10. Subnational estimates for districts in Cabo Delgado province were not produced due to incomplete programme data caused by service disruptions, with large numbers of internally displaced persons and closed health facilities.

Provincial and district‐level incidence maps and district‐level prevalence maps by sex were created in QGIS 3.28.3 using Naomi estimates. Prevalence per 100 and incidence rates per 1000 HIV‐negative population (both aged 15 years and older) were mapped and displayed by standardized quintile breaks of the referenced population. Associated 95% uncertainty ranges (UR) are reported. Northern provinces include Cabo Delgado, Niassa, Nampula and Zambezia; central provinces: Tete, Manica and Sofala; and southern provinces: Inhambane, Gaza, Maputo and Maputo City.

**Figure 1:**
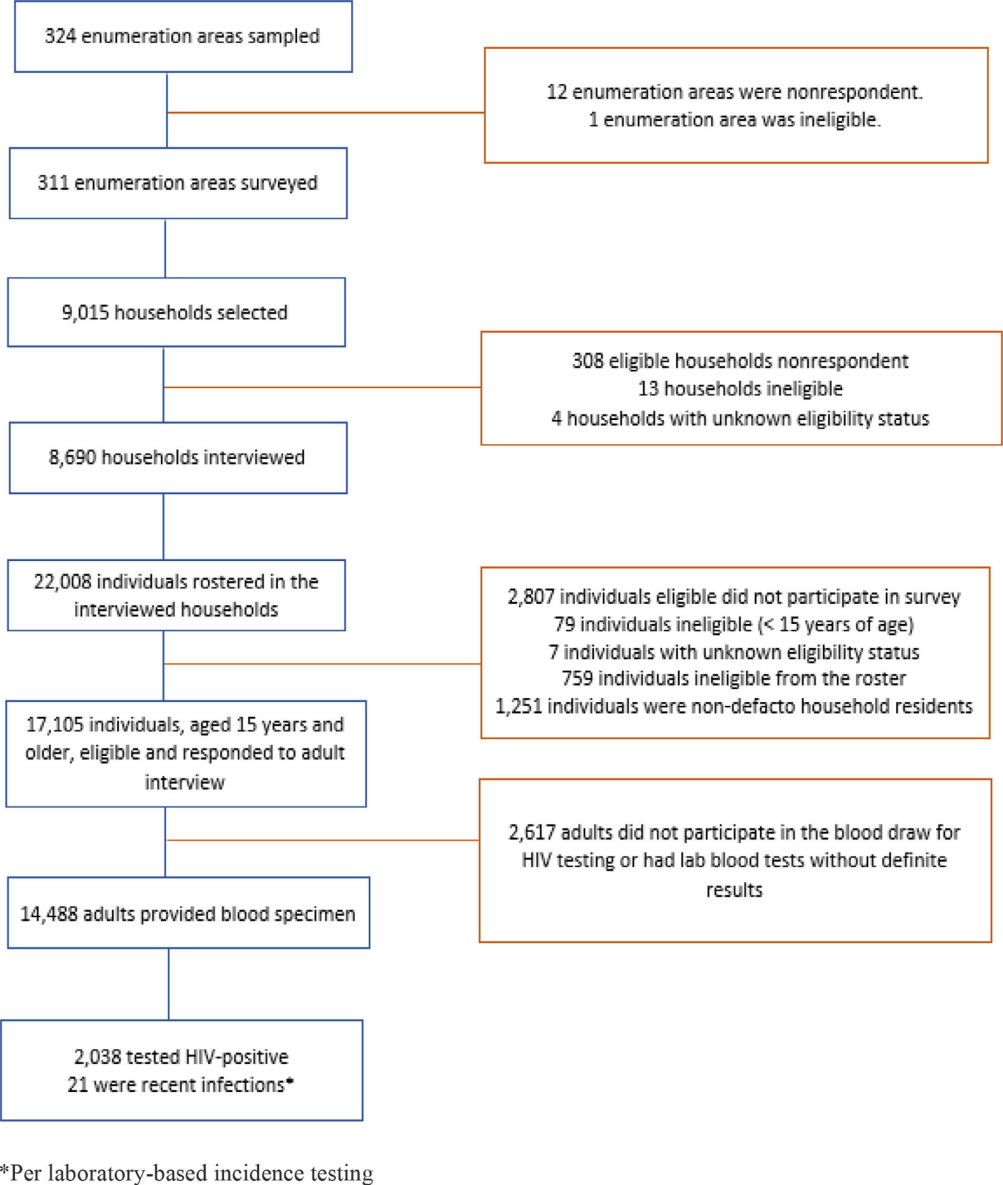
Number of EAs, households and adults—aged 15 years and older—with survey interview, blood draw, and HIV result in Mozambique, INSIDA 2021.

Analysis of risk factors associated with prevalent HIV infection was restricted to INSIDA 2021 survey participants with definite laboratory results and final HIV status (*N* = 14,488) (Figure 1). Descriptive analyses were conducted for HIV prevalence by sex and select demographic and behavioural factors, with prevalence and corresponding 95% confidence intervals (CIs) reported. All variables, except age at first sex, were treated as categorical variables. Regression analyses were restricted to participants who reported having ever had sexual intercourse. Bivariate logistic regression models were fit to analyse factors associated with HIV infection among all survey participants with a valid HIV test result, stratified by sex. Odds ratios (OR), 95% CIs and *p*‐values were reported. Variables were added in a stepwise manner, retaining those associated with HIV positivity at the 5% significance level, until the best model was identified. Adjusted ORs (aOR), 95% CIs and *p*‐values were provided. To account for survey design, analyses were weighted via the jackknife replication method of variance estimation. Sampling weights were computed to adjust for selection probability and non‐response, and post‐stratification addressed non‐coverage [11]. Data were analysed using SAS 9.4.1 (SAS Institute Inc., Cary, North Carolina, USA).

Mozambique's National Bioethics Committee (CNBS), the Columbia University Medical Center, and the U.S. Centers for Disease Control and Prevention (CDC) IRBs (See 45 C.F.R. part 46.114; 21 C.F.R. part 56.114) reviewed and approved the INSIDA survey protocol. All participants provided written consent prior to the interview and blood draw.

## 3 RESULTS

In the 8690 houses interviewed, 22,008 individuals were rostered, of which 17,105 were eligible and interviewed (85.9%, unweighted response rate), 14,488 provided a blood sample (84.7%, unweighted response rate), of whom 2038 tested HIV positive, with 21 classified as recent infections (Figure 1).

### 3.1 HIV prevalence at national and provincial levels: results from INSIDA 2021

Among adults 15 years and older in Mozambique, HIV prevalence was 12.5% (95% CI: 11.5−13.4), higher among women (15.0%; 95% CI: 13.9−16.1) than men (9.5%; 95% CI: 8.5−10.6), and highest among those aged 35–49 years (21.4%; 95% CI: 19.3−23.5) (Table 1). Among women aged 15–24 years, prevalence was over three‐fold higher (8.0%; 95% CI: 6.6−9.3) than among men the same age (2.6%; 95% CI: 1.8−3.4). Prevalence was higher in urban areas (14.2%; 95% CI 12.6−15.8) than rural (11.4%; 95% CI: 9.8−12.9) and varied considerably by province, ranging from 7.9% (95% CI: 6.0−9.8) in Manica to 20.9% (95% CI: 16.7−25.0) in Gaza. Overall, prevalence was higher in southern provinces. HIV prevalence was lowest among those who completed more than a secondary education level, 7.8%; (95% CI: 4.6−11.0), and among those in the two lowest household wealth quintiles, 9.1% (95% CI: 7.2−11.0) in the lowest and 8.5% (95% CI: 6.8−10.3) in the second lowest, with these differences especially evident among women.

**Table 1:** Prevalence of HIV among adults—aged 15 years and older—by sex and select socio‐demographic and behavioral characteristics, INSIDA 2021.

Characteristic	Men			Women			Total		
	%	95% CI	*N*	%	95% CI	*N*	%	95% CI	*N*
**Age group (years)**									
15−24	2.6	1.8−3.4	1981	8.0	6.6−9.3	2498	5.4	4.5−6.2	4479
25−34	11.0	8.5−13.5	1357	17.9	15.7−20.1	2050	14.7	13.0−16.5	3407
35−49	17.9	15.0−20.9	1569	24.4	21.6−27.3	1988	21.4	19.3−23.5	3557
50+	12.3	10.1−14.5	1326	13.3	11.5−15.2	1719	12.9	11.2−14.5	3045
**Residence**									
Urban	10.4	8.4−12.4	2429	17.7	15.9−19.4	3362	14.2	12.6−15.8	5791
Rural	9.0	7.4−10.6	3804	13.4	11.7−15.1	4893	11.4	9.8−12.9	8697
**Province**									
Niassa	5.7	3.0−8.4	540	10.3	7.0−13.6	586	8.0	5.3−10.7	1126
Cabo Delgado	6.1	3.0−9.1	412	14.3	10.4−18.2	479	10.5	7.2−13.8	891
Nampula	7.7	4.9−10.5	1044	12.3	9.4−15.1	1076	10.0	7.4−12.5	2120
Zambezia	16.3	12.3−20.4	455	17.7	12.7−22.8	567	17.1	13.0−21.2	1022
Tete	5.8	3.9−7.7	813	10.9	9.0−12.7	1017	8.4	6.7−10.1	1830
Manica	7.7	5.6−9.9	546	8.0	5.6−10.5	731	7.9	6.0−9.8	1277
Sofala	10.1	6.2−14.1	586	15.8	12.6−19.0	788	13.2	9.9−16.4	1374
Inhambane	9.3	6.3−12.2	366	14.6	11.9−17.4	743	12.6	10.5−14.7	1109
Gaza	13.0	8.3−17.7	310	25.9	21.3−30.6	640	20.9	16.7−25.0	950
Maputo Province	11.4	7.7−15.0	635	18.9	16.5−21.3	888	15.4	13.3−17.5	1523
Maputo City	11.6	9.1−14.2	526	20.2	17.3−23.2	740	16.2	13.7−18.7	1266
**Household wealth quintile**									
Lowest	5.9	3.8−8.1	910	11.4	9.3−13.6	1287	9.1	7.2−11.0	2197
Second	7.2	5.0−9.4	1223	9.9	8.1−11.6	1389	8.5	6.8−10.3	2612
Middle	11.0	8.8−13.2	1209	16.6	13.8−19.5	1450	13.9	11.9−16.0	2659
Fourth	13.1	10.7−15.5	1259	18.1	16.1−20.1	1868	15.8	13.9−17.7	3127
Highest	9.5	7.8−11.3	1614	17.5	15.4−19.6	2236	13.7	12.1−15.3	3850
**Education**									
No education	8.7	6.7−10.7	758	14.8	12.7−16.9	2353	13.2	11.4−15.0	3111
Primary	10.6	9.1−12.1	3076	16.0	14.4−17.6	3506	13.3	12.0−14.7	6582
Secondary	8.6	7.3−9.9	2150	14.2	12.4−16.0	2160	11.1	9.8−12.4	4310
More than secondary	7.4	2.3−12.4	236	8.4	4.9−11.9	213	7.8	4.6−11.0	449
**Marital status**									
Never married	2.9	2.0−3.7	1961	9.3	7.6−10.9	1656	5.5	4.6−6.4	3617
Married or living together	12.9	11.4−14.4	3673	14.0	12.7−15.3	4556	13.5	12.2−14.8	8229
Divorced or separated	15.2	11.4−19.1	479	24.9	22.0−27.8	1144	21.7	19.1−24.2	1623
Widowed	19.9	10.1−29.7	111	21.2	18.0−24.5	882	21.1	17.9−24.2	993
**Pregnancy status**									
Currently pregnant	NA		NA	10.6	7.5−13.8	470	NA		NA
Not currently pregnant	NA		NA	15.3	14.2−16.4	7706	NA		NA
**Number of lifetime sexual partners**									
0	2.7	0.9−4.4	377	2.1	0.5−3.7	358	2.4	1.3−3.6	735
1	5.5	3.7−7.3	942	9.5	8.1−10.9	3124	8.4	7.2−9.7	4066
2+	9.7	8.4−11	4032	20.5	19.0−22.0	4078	14.7	13.6−15.9	8110
**Age (years) at first sexual intercourse**									
Under 15	5.2	3.7−6.8	763	16.8	13.8−19.7	934	11.2	9.6−12.9	1697
15−19	9.5	7.9−11.0	3390	15.6	14.4−16.8	5146	12.9	11.8−14.0	8536
20−24	13.5	10.2−16.9	755	19.6	15.4−23.8	600	16.0	13.2−18.9	1355
25+	15.3	8.8−21.7	227	25.8	14.6−37.0	81	18.0	12.6−23.4	308
**Number of sexual partners in the 12 months before the survey**									
0	8.4	6.4−10.5	796	19.4	16.9−21.8	1510	15.0	13.1−16.8	2306
1	10.0	8.7−11.4	3403	14.8	13.5−16.1	5385	12.9	11.7−14.0	8788
2+	10.4	8.3−12.6	1313	22.6	17.0−28.3	291	12.5	10.3−14.7	1604
**Condom use at last sexual intercourse in the 12 months before the survey**									
Used condom	9.1	7.2−11.1	1135	20.9	18.2−23.6	970	14.0	12.2−15.8	2105
Did not use condom	10.5	9.0−11.9	3567	14.1	12.9−15.4	4680	12.5	11.2−13.7	8247
No sexual intercourse in the 12 months before the survey	8.4	6.4−10.5	796	19.4	16.9−21.8	1510	15.0	13.1−16.8	2306
**Mean age (years) at first sexual intercourse**									
	17.9	17.6−18.2		16.7	16.5−16.9		17.1	16.9−17.3	
**Total 15+**	**9.5**	**8.5**−**10.6**	**6233**	**15.0**	**13.9**−**16.1**	**8255**	**12.5**	**11.5**−**13.4**	**14,488**

*Note*: Percentages and confidence intervals are weighted and adjusted to account for the survey design.

### 3.2 Factors associated with HIV infection: results from INSIDA 2021

We assessed factors associated with HIV infection among HIV‐positive adults (*N* = 2038) who had ever had sexual intercourse (*N* = 1995), stratified by sex (Tables 2 and 3). In bivariate analysis, the odds of HIV were lower among both women and men in the youngest age group compared to older age groups (OR men: 0.12; 95% CI: 0.08−0.18; OR women: 0.31; 95% CI: 0.25−0.39). Women residing in urban areas had higher odds of being HIV positive (OR: 1.45; 95% CI: 1.18−1.77) compared to those residing in rural areas. Both men and women who initiated sex at 25 years of age or older had greater odds of being HIV positive (OR men: 1.73; 95% CI: 1.05−2.83 and OR women: 1.88; 95% CI: 1.08−3.27) than those sexually active at 15–19 years. Women currently pregnant were less likely to be HIV positive (OR: 0.62; 95% CI: 0.45−0.85) than non‐pregnant women. Men who reported condom use at last sexual intercourse were less likely to be HIV positive (OR: 0.86; 95% CI: 0.66−1.11) compared to men who did not use a condom, but odds of being HIV positive were higher among women who used a condom (OR: 1.61; 95% CI: 1.36−1.90) compared to women who did not.

**Table 2:** Factors associated with prevalent HIV infection among HIV‐positive men—15 years and older—who have ever had sex (with valid blood test results) in Mozambique, INSIDA 2021.

Men							
		Unadjusted ORs			Adjusted ORs		
Characteristic	*N* (weighted % HIV positive)	OR (95% CI)	*p*‐value	Global *p*‐value	aOR (95% CI)	*p*‐value	Global *p*‐value
**Age group (years)**							
15−24	42 (2.6)	0.12 (0.08−0.18)	< 0.001	<0.001	0.18 (0.12−0.28)	<0.001	<0.001
25−34	127 (10.9)	0.57 (0.41−0.78)	0.069		0.61 (0.45−0.84)	0.161	
35−49	285 (17.8)	ref	ref		ref	ref	
50+	157 (12.1)	0.63 (0.50−0.81)	0.000		0.65 (0.51−0.84)	0.035	
**Residence**							
Urban	267 (10.8)	1.14 (0.84−1.54)	0.406	0.407			
Rural	344 (9.6)	ref	ref				
**Province**							
Niassa	32 (5.8)	0.66 (0.35−1.25)	0.199	<0.001	0.67 (0.35−1.29)	0.102	<0.001
Cabo Delgado	24 (6.1)	0.70 (0.36−1.38)	0.298		0.75 (0.39−1.44)	0.241	
Nampula	87 (8.5)	ref	ref		ref	ref	
Zambezia	80 (16.3)	2.11 (1.28−3.46)	0.003		2.14 (1.27−3.59)	<0.001	
Tete	46 (5.9)	0.68 (0.40−1.14)	0.139		0.49 (0.27−0.89)	<0.001	
Manica	51 (8.6)	1.02 (0.60−1.72)	0.952		0.79 (0.45−1.37)	0.111	
Sofala	50 (11.4)	1.39 (0.77−2.48)	0.268		1.25 (0.67−2.32)	0.268	
Inhambane	42 (10.2)	1.23 (0.73−2.10)	0.433		1.13 (0.63−2.01)	0.571	
Gaza	52 (14.6)	1.85 (1.05−3.28)	0.033		1.55 (0.81−2.94)	0.045	
Maputo Province	75 (12.1)	1.49 (0.88−2.53)	0.139		0.97 (0.53−1.77)	0.803	
Maputo City	72 (11.9)	1.46 (0.91−2.35)	0.117		1.26 (0.66−2.41)	0.246	
**Household wealth quintile**							
Lowest	52 (6.4)	0.62 (0.39−0.99)	0.043	<0.001	0.47 (0.26−0.86)	0.002	<0.001
Second	89 (7.5)	0.74 (0.50−1.08)	0.112		0.60 (0.35−1.02)	0.045	
Middle	134 (11.5)	1.17 (0.87−1.57)	0.285		0.84 (0.55−1.29)	0.649	
Fourth	164 (14.0)	1.48 (1.15−1.89)	0.002		1.38 (1.03−1.86)	<0.001	
Highest	172 (9.9)	ref	ref		ref	ref	
**Education**							
No education	68 (8.6)	0.75 (0.55−1.03)	0.075	0.127	0.74 (0.52−1.05)	0.922	0.079
Primary	340 (11.1)	ref	ref		ref	ref	
Secondary	184 (9.3)	0.82 (0.67−1.00)	0.045		0.90 (0.71−1.14)	0.118	
More than secondary	17 (7.3)	0.63 (0.30−1.29)	0.203		0.48 (0.24−0.95)	0.082	
**Marital status**							
Never married	53 (3.0)	0.21 (0.15−0.30)	<0.001	<0.001	0.41 (0.25−0.67)	<0.001	<0.001
Married/Living together	465 (12.7)	ref	ref		ref	ref	
Divorced/Separated	69 (15.3)	1.25 (0.94−1.66)	0.119		1.24 (0.93−1.65)	0.157	
Widowed	23 (21.9)	1.93 (1.03−3.63)	0.040		2.11 (1.03−4.34)	0.008	
**Male circumcision**							
Fully or partially circumcised	364 (8.5)	ref	ref	<0.001	ref	ref	0.008
Uncircumcised	244 (14.2)	1.79 (1.37−2.33)	<0.001		1.54 (1.12−2.10)	0.007	
**Age (year) at first sexual intercourse**							
Under 15	50 (5.2)	0.53 (0.36−0.78)	0.001	<0.001			
15−19	333 (9.5)	ref	ref				
20−24	112 (13.5)	1.50 (1.06−2.12)	0.020				
25+	33 (15.3)	1.73 (1.05−2.83)	0.029				
**Number of sexual partners in the 12 months before the survey**							
None	72 (8.1)	0.82 (0.63−1.07)	0.140	0.309			
1	372 (10.0)	ref	ref				
2 or more	143 (10.4)	1.04 (0.83−1.32)	0.713				
**Condom use at last sexual intercourse in the 12 months before the survey**							
Did not have sex	72 (8.4)	0.79 (0.60−1.03)	0.083	0.166	0.98 (0.73−1.30)	0.108	0.022
Did not use a condom	373 (10.5)	ref	ref		ref	ref	
Used a condom	139 (9.1)	0.86 (0.66−1.11)	0.245		1.53 (1.11−2.09)	0.006	

*Note*: Percentages and confidence intervals are weighted and adjusted to account for the survey design. Global *p*‐value refers to the overall level of statistical significance for the independent categorical variable. Respondents with missing values were excluded from tabulations (11 men reported never having sex; 20 men had unknown/missing values).

Abbreviations: aOR, adjusted odds ratio; CI, confidence interval; OR, odds ratio.

**Table 3:** Factors associated with prevalent HIV infection among HIV‐positive women—15 years and older—who have ever had sex (with valid blood test results) in Mozambique, INSIDA 2021.

Women							
		Unadjusted ORs			Adjusted ORs		
Characteristic	*N* (weighted % HIV positive)	OR (95% CI)	*p*‐value	Global *p*‐value	aOR (95% CI)	*p*‐value	Global *p*‐value
**Age group (years)**							
15−24	184 (9.1)	0.31 (0.25−0.39)	<0.001	<0.001	0.29 (0.22−0.38)	<0.001	<0.001
25−34	388 (17.9)	0.67 (0.55−0.83)	0.004		0.69 (0.55−0.85)	<0.001	
35−49	546 (24.4)	ref	ref		ref	ref	
50+	266 (13.4)	0.48 (0.40−0.57)	0.018		0.40 (0.32−0.51)	0.003	
**Residence**							
Urban	679 (19.0)	1.45 (1.18−1.77)	<0.001	<0.001	1.27 (0.93−1.75)	0.133	0.135
Rural	705 (13.9)	ref	ref		ref	ref	
**Province**							
Niassa	70 (10.7)	0.83 (0.53−1.29)	0.399	<0.001	1.00 (0.63−1.6)	0.099	<0.001
Cabo Delgado	72 (14.5)	1.18 (0.78−1.76)	0.429		1.41 (0.95−2.08)	0.702	
Nampula	135 (12.6)	ref	ref		ref	ref	
Zambezia	106 (18.1)	1.53 (1.01−2.32)	0.045		1.84 (1.22−2.78)	0.029	
Tete	116 (11.3)	0.88 (0.64−1.22)	0.438		0.92 (0.66−1.29)	<0.001	
Manica	72 (8.8)	0.67 (0.44−1.01)	0.057		0.74 (0.49−1.12)	<0.001	
Sofala	124 (17.2)	1.44 (1.00−2.06)	0.046		1.81 (1.28−2.56)	0.002	
Inhambane	125 (15.7)	1.29 (0.92−1.82)	0.137		1.26 (0.82−1.93)	0.663	
Gaza	195 (27.4)	2.62 (1.83−3.75)	<0.001		2.64 (1.75−3.99)	<0.001	
Maputo Province	199 (20.6)	1.80 (1.32−2.44)	<0.001		1.49 (1.02−2.16)	0.299	
Maputo City	170 (21.7)	1.92 (1.39−2.64)	<0.001		1.52 (1.03−2.24)	0.324	
**Household wealth quintile**							
Lowest	152 (11.8)	0.57 (0.43−0.74)	<0.001	<0.001	0.69 (0.40−1.18)	0.097	0.006
Second	146 (10.1)	0.48 (0.38−0.60)	<0.001		0.64 (0.41−1.01)	0.008	
Middle	254 (17.1)	0.88 (0.69−1.12)	0.282		1.01 (0.7−1.45)	0.086	
Fourth	379 (19.2)	1.01 (0.83−1.22)	0.941		1.15 (0.88−1.49)	0.003	
Highest	451 (19.1)	ref	ref		ref		
**Education**							
No education	363 (14.9)	0.87 (0.72−1.06)	0.159	0.015	0.94 (0.78−1.15)	0.004	<0.001
Primary	667 (16.7)	ref	ref		ref	ref	
Secondary	329 (15.8)	0.94 (0.79−1.12)	0.461		0.82 (0.67−0.99)	0.055	
More than secondary	22 (8.8)	0.48 (0.31−0.76)	0.002		0.32 (0.19−0.52)	<0.001	
**Marital status**							
Never married	157 (11.7)	0.81 (0.66−1.00)	0.053	<0.001	0.95 (0.73−1.24)	0.001	<0.001
Married/Living together	691 (14.0)	ref	ref		ref	ref	
Divorced/Separated	313 (24.9)	2.04 (1.72−2.43)	<0.001		1.94 (1.55−2.42)	<0.001	
Widowed	219 (21.3)	1.67 (1.37−2.02)	<0.001		1.79 (1.37−2.34)	0.007	
**Pregnancy status**							
Currently pregnant	59 (10.6)	0.62 (0.45−0.85)	0.003	0.004			
Not currently pregnant	1308 (16.1)	ref	ref				
**Age (years) at first sexual intercourse**							
Under 15	176 (16.8)	1.09 (0.89−1.34)	0.403	0.019			
15−19	899 (15.6)	ref	ref				
20−24	118 (19.6)	1.32 (1.02−1.72)	0.035				
25+	23 (25.8)	1.88 (1.08−3.27)	0.024				
**Number of sexual partners in the 12 months before the survey**							
None^a^	335 (19.4)	1.38 (1.17−1.63)	<0.001	<0.001			0.072
1	873 (14.8)	ref			ref	ref	
2 or more	71 (22.6)	1.68 (1.23−2.30)	0.001		1.44 (0.97−2.15)	0.071	
**Condom use at last sexual intercourse in the 12 months before the survey**							
Did not have sex	335 (19.4)	1.46 (1.22−1.74)	<0.001	<0.001	1.00 (0.74−1.36)	0.117	<0.001
Did not use a condom	706 (14.1)	ref	ref		ref		
Used a condom	235 (20.9)	1.61 (1.36−1.9)	<0.001		1.60 (1.30−1.96)	<0.001	

*Note*: Percentages and confidence intervals are weighted and adjusted to account for the survey design. Global *p*‐value refers to the overall level of statistical significance for the independent categorical variable. Respondents with missing values were excluded from tabulations (9 women reported never having sex; 3 women had unknown/missing values).

Abbreviations: aOR, adjusted odds ratio; CI, confidence interval; OR, odds ratio.

^a^ This aOR is missing due to perfect prediction between condom use and sexual partners in the last 12 months for those who did not have sex.

The final multivariable model for men was adjusted for age, province, household wealth, education, marital status, male circumcision and condom use (Table 2). The final model for women was adjusted for age, residence, province, household wealth, education, marital status, number of sexual partners and condom use (Table 3). For both sexes, the odds of prevalent HIV infection were lower in all age groups compared to those aged 35–49 years, with the youngest age group, 15–24 years, the least likely to be HIV positive (aOR men: 0.18; 95% CI: 0.12−0.28; aOR women: 0.29; 95% CI: 0.22−0.38).

In adjusted analyses, men residing in Zambezia province had over twice the odds of being HIV positive (aOR: 2.14; 95% CI: 1.27−3.59) and men in Tete had half the odds (aOR: 0.49; 95% CI: 0.27−0.89), compared to those in Nampula province. Alternatively, women in Gaza province had more than twice the odds of being HIV positive (aOR: 2.64; 95% CI: 1.75−3.99) compared to women in Nampula. The odds of being HIV positive increased with household wealth quintile in both men and women. Men in the lowest quintile had 53% (aOR: 0.47; 95% CI: 0.26−0.86) lower odds of being HIV positive, and women had 31% (aOR: 0.69; 95% CI: 0.40−1.18) lower odds compared to the highest quintile. Women with more than a secondary education level had 68% (aOR: 0.32; 95% CI: 0.19−0.52) lower odds of being HIV positive compared to those with a primary education level. Compared to those married, men never been married had 59% lower odds of being HIV positive (aOR: 0.41; 95% CI: 0.25−0.67). Both widowed men and women were significantly more likely to be HIV positive compared to those married (aOR men: 2.11; 95% CI: 1.03−4.34; aOR women: 1.79; 95% CI: 1.37−2.34). Male circumcision was significantly associated with HIV infection; uncircumcised men had 54% greater odds of being HIV positive (aOR: 1.54; 95% CI: 1.12−2.10) than those fully or partially circumcised. Condom use was associated with greater odds of being HIV positive in both men and women (aOR men: 1.53; 95% CI: 1.11−2.09; aOR women: 1.60; 95% CI: 1.30−1.96) when compared to not using a condom at last intercourse.

### 3.3 District‐level HIV prevalence: results from the Naomi Model 2023

**Figure 2:**
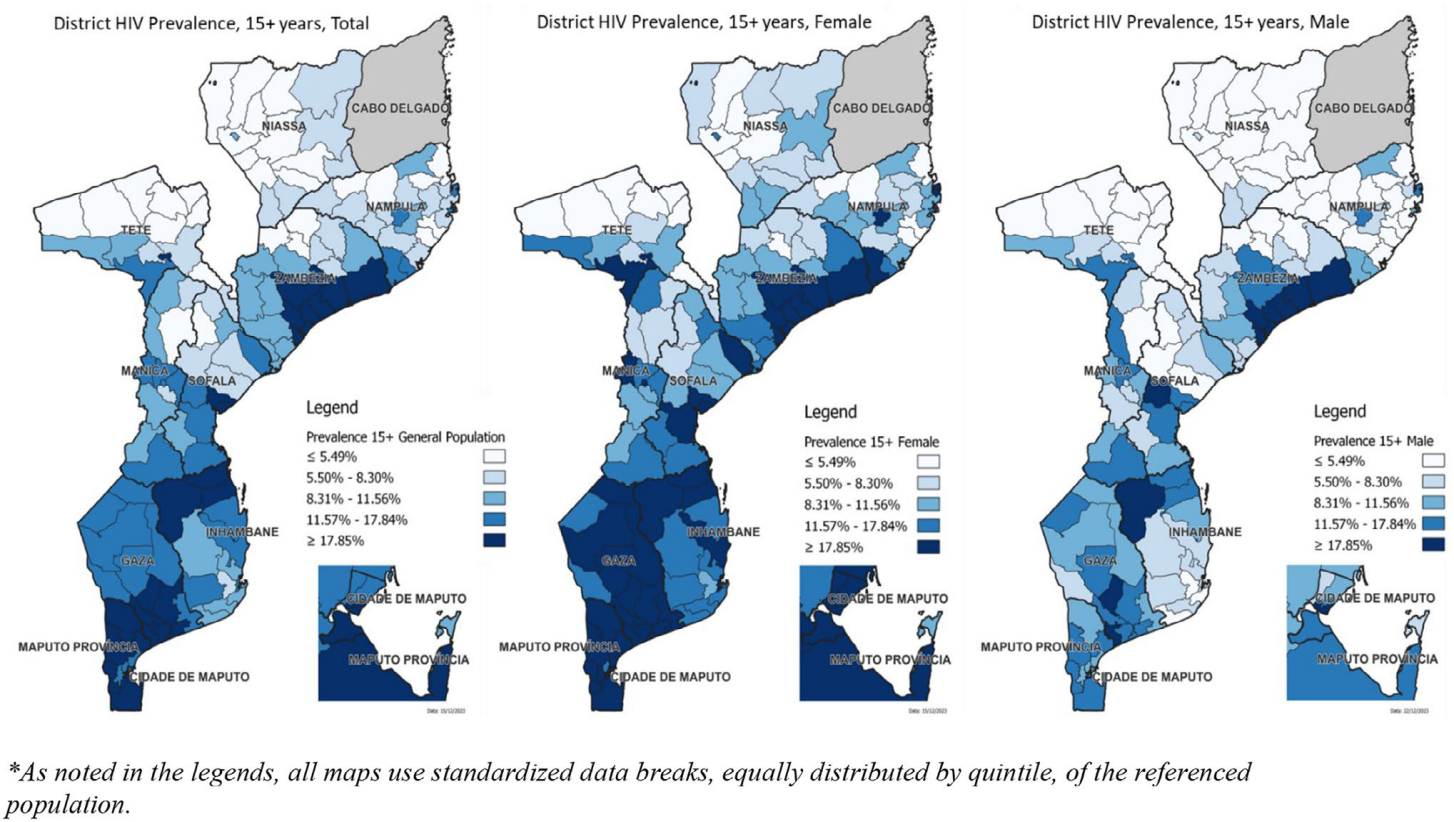
District‐level HIV prevalence among adults—aged 15 years and older—in Mozambique by sex, 2023 Naomi estimates.

Compared to other central provinces, Zambezia had disproportionately high prevalence in district‐level HIV prevalence maps (Figure 2). Adult HIV prevalence in the coastal districts of Nicoadala (28.4%; 95% Uncertainty Range [UR/95% credible interval^10^]: 22.8−34.6), Quelimane (30.0%; 95% UR: 26.5−33.6), Namacurra (25.9%; 95% UR: 20.2−32.3), Maganja da Costa (25.9%; 95% UR 19.7−32.5), Mocubela (30.1%; 95% UR: 25.1−35.3) and Pebane (26.1%; 95% UR: 21.9−30.3) were within the highest prevalence quintile across all districts, matching the high HIV prevalence in some southern districts. The district‐level maps also highlighted higher HIV prevalence in provincial capitals compared to surrounding areas and along the transportation corridor in the centre of the country. This corridor extends from the port of Beira (19.0%; 95% UR: 17.7−21.5) through Dondo (20.1%; 95% UR: 14.4−26.7) and Nhamatanda (15.3%; 95% UR: 13.7−16.9) districts in Sofala and continues through the Manica districts: Gondola (12.1%; 95% UR: 9.5−14.6), Chimoio (16.8%; 95% UR: 15.6−18.2), Vanduzi (13.8%; 95% UR: 10.8−17.5) and Manica (16.1%, 95% UR: 13.8−18.4) to the Zimbabwe border. Comparing districts by sex, HIV prevalence among women was higher than among men, particularly in the districts of the southern provinces of Gaza and Maputo.

### 3.4 National‐level HIV incidence: results from INSIDA 2021

Recent infection testing in the INSIDA 2021 survey identified 21 recent infections, estimating national HIV incidence of 4.3 per 1000 HIV‐negative adults aged 15 years and older (95% CI: 2.3−6.3), with incidence of 2.4 (95% CI: 0.2−4.6) among men and 6.1 (95% CI: 2.9−9.3) per 1000 among women.

### 3.5 Provincial and district‐level HIV incidence: results from the Naomi Model 2023

Modelled HIV incidence was highest in the southern provinces, Gaza (7.5 per 1000 population) and Maputo City (7.0 per 1000) (Table 4). Despite bordering Gaza province and Maputo City, incidence was slightly lower in Maputo Province (6.4 per 1000), similar to incidence in the central provinces of Zambezia (5.4 per 1000) and Manica (5.3 per 1000), but still higher than lower burden provinces, such as Tete and Niassa.

**Table 4:** HIV incidence (per 1000 persons) among adults—15 years and older—by province in Mozambique, Naomi Model 2023.

Province	Female		Male		Total	
	HIV incidence	95% CI	HIV incidence	95% CI	HIV incidence	95% CI
Niassa	4.30	3.7−5.0	2.30	2.0−2.7	3.30	2.8−3.9
Cabo Delgado	5.93	5.2−6.8	3.34	2.9−3.8	4.64	4.0−5.3
Nampula	5.40	4.8−6.0	2.97	2.6−3.3	4.18	3.7−4.7
Zambezia	6.98	6.3−7.7	3.69	3.3−4.1	5.36	4.8−5.9
Tete	4.11	3.7−4.6	2.29	2.1−2.6	3.19	2.9−3.6
Manica	6.93	6.3−7.6	3.68	3.4−4.0	5.30	4.8−5.8
Sofala	9.21	8.4−10.2	4.88	4.4−5.5	7.00	6.3−7.8
Inhambane	6.70	5.9−7.7	3.66	3.2−4.2	5.29	4.7−6.0
Gaza	9.61	8.7−10.6	5.16	4.7−5.7	7.52	6.8−8.3
Maputo Province	8.27	7.3−9.3	4.57	4.1−5.1	6.37	5.6−7.1
Maputo City	9.18	8.1−10.5	5.09	4.5−5.8	7.01	6.2−8.0

**Figure 3:**
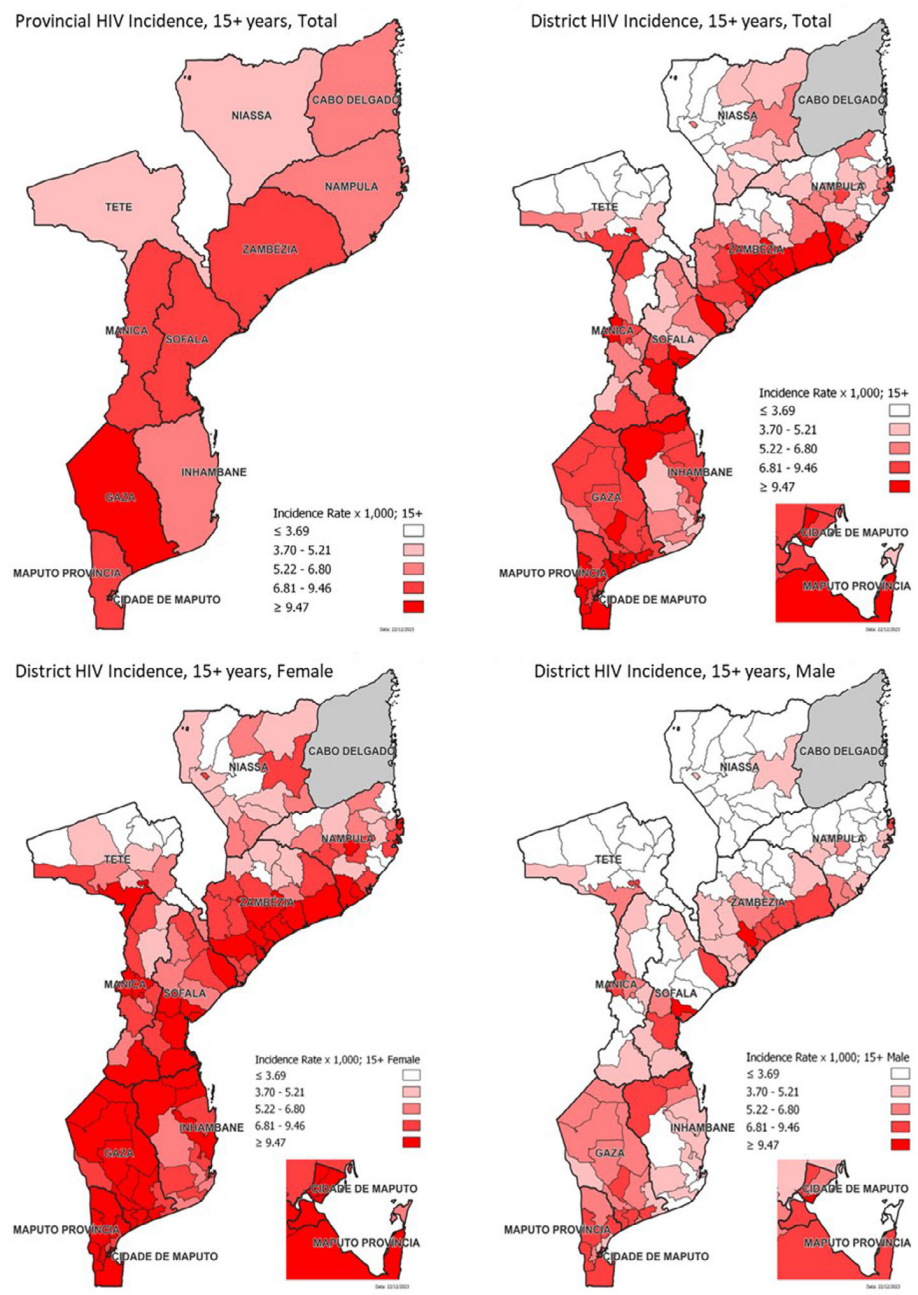
Provincial (for both sexes) and district‐level incidence among adults—aged 15 years and older—by sex in Mozambique, per 1000 people, Naomi Model 2023. Note: *As noted in the legends, all maps use standardized data breaks, equally distributed by quintile, of the referenced population.

Modelled HIV incidence estimates by district were highest in the Nicoadala district of Zambezia province (16.0; 95% UR: 11.7−20.9), though with large uncertainty ranges (Figure 3). Similar to district prevalence estimates, HIV incidence was also higher in the costal districts of Zambezia, including Nicoadala, Mocubela (14.0; 95% UR: 11.0−17.7), Quelimane (13.5; 95% UR: 11.0−16.6), Maganja da Costa (13.5; 95% UR: 9.6−18.4), Pebane (12.5; 95% UR: 9.9−15.4) and Namacurra (12.3; 95% UR: 9.0−16.7), all with incidence rates greater than 12.0 per 1000 persons. Districts along the coast of Gaza Province, Maputo Province and Maputo City had high incidence rates as well, above 9.0 per 1000 persons. Higher incidence was also noted in nearly all provincial capital cities, except Lichinga in Niassa province (5.6 per 1000; 95% UR: 4.27−7.27). As noted in the provincial HIV incidence map, southern districts had a higher modelled incidence, followed by the central, then northern provinces; however, more heterogeneity was observed at the district level, particularly among women.

### 3.6 Estimated number of people living with HIV and new HIV infections: results from the Naomi Model 2023

Model results estimated that roughly 2.2 million people (95% CI: 2,200,000−2,300,000) aged 15 years and older were living with HIV in Mozambique in 2023, with nearly twice as many women (1,460,000) than men (810,000) living with HIV (Table 4). The estimated number of PLHIV varied by province, from 72,000 in Niassa to 450,000 in Zambezia.

There were an estimated 84,000 (95% CI: 80,000−89,000) new HIV infections among adults aged 15 years and older in 2023, 30,000 (95% CI: 28,000−31,000) among men and 55,000 (95% CI: 52,000−58,000) among women (Table 5). Similar to the findings for PLHIV, the number of new HIV infections was largest in Zambezia, 15,000 (95% CI: 13,800−16,600), and lowest in Niassa, 3400 (95% CI: 2900−4000).

**Table 5:** Number of people living with HIV and new HIV infections among adults—aged 15 years and older—per sex and province, in Mozambique, Naomi Model 2023.

Province	Number of PLHIV						Number of new HIV infections					
	Female		Male		Total		Female		Male		Total	
	*N*	95% CI	*N*	95% CI	*N*	95% CI	*N*	95% CI	*N*	95% CI	*N*	95% CI
Niassa	44,000	41,000−48,000	25,000	22,000−28,000	69,000	63,000−76,000	2200	1900−2600	1200	1000−1400	3400	2900−4000
Cabo Delgado	87,000	80,000−94,000	56,000	50,000−62,000	140,000	130,000−150,000	4300	3700−4900	2400	2100−2700	6700	5800−7600
Nampula	200,000	190,000−210,000	120,000	110,000−130,000	320,000	300,000−340,000	8900	8000−9900	5000	4400−5500	13,900	12,400−15,400
Zambézia	270,000	260,000−280,000	180,000	170,000−190,000	450,000	430,000−460,000	10,000	9100−11,000	5100	4700−5600	15,100	13,800−16,600
Tete	76,000	73,000−80,000	48,000	44,000−52,000	120,000	120,000−130,000	3300	2900−3600	1900	1700−2100	5100	4600−5700
Manica	96,000	92,000−100,000	55,000	52,000−58,000	150,000	140,000−160,000	3800	3500−4200	2000	1800−2200	5800	5400−6300
Sofala	140,000	130,000−140,000	90,000	80,000−90,000	220,000	210,000−240,000	6000	5500−6500	3300	3000−3700	9300	8500−10,200
Inhambane	94,000	89,000−100,000	34,000	30,000−37,000	130,000	120,000−140,000	3400	3000−3800	1600	1400−1800	5000	4400−5600
Gaza	150,000	140,000−150,000	63,000	60,000−67,000	210,000	200,000−220,000	4000	3700−4400	1900	1800−2100	5900	5500−6500
Maputo Province	160,000	150,000−170,000	85,000	78,000−90,000	240,000	230,000−260,000	5300	4700−5900	3100	2800−3400	8400	7500−9300
Maputo City	120,000	110,000−120,000	59,000	55,000−60,000	170,000	160,000−180,000	3400	3100−3900	2200	1900−2400	5600	5000−6300
Mozambique^a^	1,400,000	1,400,000−1,500,000	800,000	780,000−830,000	2,200,000	2,200,000−2,300,000	55,000	52,000−58,000	30,000	28,000−31,000	84,000	80,000−89,000

^a^ Columns may not sum to the national total due to rounding.

## 4 DISCUSSION

The INSIDA 2021 survey reported 12.5% national HIV prevalence among adults aged 15 years and older in Mozambique, with the Naomi Model, estimating 2.2 million PLHIV and 84,000 new infections in 2023. Among adults aged 15–49 years, prevalence was 12.4% in 2021, slightly lower than 13.2% in 2015, with 4.3 new infections per 1000 people compared to 6.0 in 2015 [12, 13]. Declining HIV prevalence, consistent with reductions in new HIV infections, aligns with HIV prevention and treatment scale‐up in Mozambique over the past decade [14]. Despite a decrease from 160,000 annual new infections in 2010 to 84,000 in 2023, Mozambique fell short of the UNAIDS 75% reduction target for 2020, which would require fewer than 41,000 new infections [15]. Among UNAIDS Global Prevention Coalition (GPC) focus countries, Mozambique ranks low in incidence reduction [15]. Though modest compared to other countries, incidence declines in Mozambique may reflect gains in ART coverage, emphasizing the need to reinforce HIV testing, linkage and retention in care [15]. In terms of achieving the 2025 UNAIDS 95‐95‐95 targets (95% of PLHIV aware of their HIV status, 95% of those aware receiving ART, and of the later, 95% have viral load suppression [VLS]), Mozambique lags behind with 71.6%−96.4%−89.4%, respectively, per INSIDA 2021 data [12]. Overall population VLS among all PLHIV was only 64.1% [12], indicating the need to identify all PLHIV, expand ART coverage, and achieve widespread VLS to lower HIV transmission risk and optimize treatment benefits.

Despite overall progress, HIV remains disproportionally concentrated among women in Mozambique [13, 16, 17, 18, 19]. Compared to men, prevalence and incidence were substantially higher among women [20]. Among young females aged 15–24 years, prevalence was roughly three‐fold higher than among young males. Mozambique is among the seven GPC focus countries with the greatest sex disparity in reducing new infections [15]. While adolescent girls and young women (AGYW) comprise less than 10% of the population, they represent 29% of new infections, highlighting the importance of comprehensive sex education, access to sexual and reproductive health, increased ART coverage among men and youth‐centred HIV‐prevention programmes, including pre‐exposure prophylaxis, at health facilities, schools and within communities [4]. Though this analysis focused on adult prevalence, other spatial analyses showed areas of high prevalence among young adults mirrored those of adults [21]. Thus, HIV‐prevention programmes reaching young adults, particularly AGYW, in areas of high adult HIV prevalence may consequently impact the HIV burden among young adults.

Mozambique, like other sub‐Saharan African countries, faces a geographically heterogeneous HIV epidemic, making district‐level HIV estimates from the Naomi model essential to focus interventions in high‐incidence areas and among populations at risk of HIV exposure [22, 23]. Findings revealed elevated incidence and prevalence in Maputo City and provincial capitals, consistent with studies demonstrating an association between HIV and population density or economic activity, such as urban centres, large cities or along major highways [10, 17, 18, 21]. Subnational mapping also pinpointed higher incidence and prevalence pockets along the Zambezia coastline, north of the provincial capital Quelimane, and along the Beira port‐Zimbabwe transportation route, supported by previous findings [21]. Coastal Zambezia and southern Mozambique maintained high prevalence, with the latter linked to border crossings and labour migration to South Africa [21, 22, 23, 24]. Notably, Zambezia experienced an increase in prevalence from 15.1% in 2015 to 18.2% in 2021, reinforcing the need for further research and tailored interventions [4, 12, 13].

Higher HIV prevalence was associated with greater wealth and lack of male circumcision, per INSIDA 2021 findings. VMMC has been prioritized in Mozambique since 2013 and is encouraged as part of combination HIV‐prevention programmes in nations with large HIV epidemics [25]. The relationship between wealth and risk of HIV exposure is inconsistent [26, 27], with some studies indicating a higher likelihood of HIV among wealthier individuals, despite their greater access to HIV information and services [17, 20, 26]. Both men and women reporting condom use during their last sexual intercourse had higher odds of being HIV positive. Studies demonstrate associations between condom use and riskier sexual behaviour, with higher HIV prevalence among reported condom users in Rwanda [21, 28]. Another study found HIV‐positive women significantly less likely than men to report condom use, highlighting the challenges women experience in negotiating condom use [29]. This counterintuitive finding may reflect the survey's cross‐sectional design, with condom use reported at a single time point, while HIV is a long‐term condition. Some individuals may have adopted condom use after diagnosis, or older age groups, with higher HIV prevalence, are more likely to report use. Strengthening programmes promoting condom use and empowering women to negotiate its use could help reduce new infections.

Despite providing novel insights by combining survey data with modelling and minimizing biases through using representative survey data with standardized questionnaires and measurements, several limitations should be considered. First, self‐reported survey responses—condom use, number of sexual partners and age at first intercourse—could be influenced by recall or social desirability bias. Second, there is uncertainty in routinely reported antenatal service delivery data and ART programme data, critical surveillance data inputs to the Naomi model [30]. Additionally, service disruptions and internally displaced persons in Cabo Delgado potentially affected HIV prevalence and service delivery. Lastly, wide uncertainty around survey HIV prevalence estimates in Zambezia, Mozambique's second‐most populous province, could have resulted in misestimation of the number of PLHIV.

## 5 CONCLUSIONS

Substantial expansion of HIV testing, prevention and treatment services in Mozambique has contributed to decreases in incidence and the number of annual new infections over the past decade; however, Mozambique still lags behind other East and Southern African countries in progress towards achieving the UNAIDS target of reducing new infections 82.5% by 2025 [15]. Closing the gender gap in new infections is critically important, necessitating focus on women‐centred HIV prevention programmes that include social and economic support, and increased ART coverage among men. Given the heightened risk of HIV acquisition among AGYW, implementing youth‐specific services and HIV‐exposure mitigation programmes among youth, particularly girls, in health facilities, schools and other community settings could help reach more AGYW. With provincial and district‐level findings highlighting high HIV prevalence and incidence pockets in urban areas, port cities, coastal Zambezia and southern Mozambique, focusing HIV resources and interventions in these areas could more effectively reduce the HIV burden. Ultimately, expanding HIV testing to close the gap in awareness of HIV status, along with providing ART and supporting adherence, is key to reducing the risk of HIV transmission. Additionally, addressing barriers to care and treatment, including stigma, discrimination and gender inequality, that inhibit the impact of these programmes, remains important. This focused approach, combined with effective primary prevention and continued political commitment, could help advance Mozambique's goal of ending HIV as a public health threat.

## COMPETING INTERESTS

JWI‐E is a Deputy Editor of the Journal of the International AIDS Society. No other competing interests were declared.

## AUTHORS’ CONTRIBUTIONS

EF conceived the study. DC, JWI‐E, OS, OT and MB conducted the analyses. KCM, EF and OT drafted the manuscript. ESG, MB, JM, LM and WME‐S provided essential subject matter expertise. All authors contributed to the critical review of the manuscript.

## FUNDING

The 2021 Mozambique Population‐based HIV Impact Assessment (INSIDA 2021) was funded by the President's Emergency Plan for AIDS Relief (PEPFAR) through the Centers for Disease Control and Prevention (CDC) under the terms of cooperative agreement award #U2GGH002173. OS and JWI‐E acknowledge funding from UNAIDS, the Bill & Melinda Gates Foundation (INV‐006733) and the MRC Centre for Global Infectious Disease Analysis (reference MR/X020258/1), funded by the UK Medical Research Council (MRC). This UK‐funded award is carried out in the frame of the Global Health EDCTP3 Joint Undertaking. The findings and conclusions in this report are those of the authors and do not necessarily represent the official position of the funding agencies.

For the purpose of open access, the authors have applied a Creative Commons Attribution (CC BY) license to any Author Accepted Manuscript version arising.

## Data Availability

The 2021 INSIDA public release datasets and files are available at the ICAP PHIA website (https://phia‐data.icap.columbia.edu/datasets?country_id = 15). Survey data use manuals and documentation are available for download. For access to datasets, please register for an account and submit a data request form.
